# Association of HPV in the Genesis of Head and Neck Squamous Cell Carcinoma: A Case–Control Study in a Bulgarian Cohort

**DOI:** 10.3390/cancers17172907

**Published:** 2025-09-04

**Authors:** Elitsa Deliverska, Vessela Raykova, Stanislav Yordanov, Daniel Markov, Svetoslav Slavkov, Viktor Lenkov, Zdravka Pashova-Tasseva

**Affiliations:** 1 Department of Dental, Oral and Maxillofacial Surgery, Faculty of Dental Medicine, Medical University of Sofia, 1431 Sofia, Bulgaria; elitsa.deliverska@fdm.mu-sofia.bg; 2 Department of Medical Microbiology, Medical Faculty, Medical University of Sofia, 1431 Sofia, Bulgaria; pumpi@abv.bg; 3 Department of ENT Diseases, University Hospital St. Anna, 1750 Sofia, Bulgaria; stanio_hr@icloud.com; 4 Department of Maxillofacial Surgery, Pirogov Hospital, 1606 Sofia, Bulgaria; dvmarkov@yahoo.com (D.M.); slavkov1970@gmail.com (S.S.); 5 Faculty of Dental Medicine, Medical University of Sofia, 1431 Sofia, Bulgaria; viktorlenkovv@gmail.com; 6 Department of Periodontology, Faculty of Dental Medicine, Medical University of Sofia, 1431 Sofia, Bulgaria

**Keywords:** head and neck squamous cell carcinoma (NHSCC), human papillomavirus (HPV), oropharyngeal squamous cell carcinoma (OPSCC), diagnostics, risk factors

## Abstract

**Background/Objectives:** Head and neck squamous cell carcinoma (NHSCC) is a significant global health burden, with human papillomavirus (HPV) recognized as a major etiological factor in a growing proportion of cases. The interaction between HPV status, tumor characteristics, and other risk factors remains an important focus for both prevention and clinical management. This study aimed to investigate the association between HPV and OPSCC in a Bulgarian cohort, with emphasis on the influence of behavioral and clinical factors, as well as basic tumor features. **Methods:** Eighty-nine participants were enrolled, including 50 patients with histologically confirmed NHSCC and 39 healthy controls. Clinical examinations and histopathological verification were conducted for all cases. Brush smear and oral rinse samples were collected for HPV testing, in line with molecular detection protocols, specifically PCR-based assays for viral DNA. Demographic data, behavioral risk factors, and information on concomitant diseases were obtained and analyzed in relation to HPV status. **Results:** Differences were identified between HPV-positive and HPV-negative NHSCC cases in relation to tumor characteristics, including clinical presentation and histological differentiation. Additional analyses demonstrated associations between certain behavioral factors, comorbidities, and the risk of NHSCC. **Conclusions:** This study provides data on HPV prevalence, related tumor features, and associated risk factors in OPSCC within a Bulgarian population. These findings highlight descriptive trends related to HPV status but indicate no robust statistical associations in this cohort.

## Simple Summary

This study examined the association between human papillomavirus (HPV) infection and head and neck squamous cell carcinoma (NHSCC) in a small Bulgarian cohort. Data were presented on the influence of various risk factors, alongside fundamental tumor characteristics in both patients and healthy controls. Statistically significant differences were identified between clinical and histopathological findings, underscoring the relevance of HPV status in relation to tumor behavior and disease profile.

## 1. Introduction

### 1.1. HPV in the Pathogenesis of Diverse Diseases Including Cancer

HPV (Human papilloma virus) is a small, nonenveloped virus with a double-stranded DNA structure that targets squamous epithelial tissues. Its genetic material encodes early elements such as E1 and E2, involved in replication; E5,E6, and E7, which act as oncogenic drivers by interfering with host cell cycle regulation; and E4, a gene expressed early in infection, though its precise role remains unclear. The late region contains L1 and L2, which produce the viral capsid components. Among these, E6 and E7 are particularly critical to malignant transformation, primarily through the suppression of key tumor suppressors—p53 (tumor protein p53) and Rb (retinoblastoma protein). Most HPV-associated oropharyngeal squamous cell carcinomas (OPSCC) are linked to the high-risk subtype HPV16, whose E6 and E7 proteins display stronger affinity for these regulatory targets compared to those of less oncogenic variants [1,2,3].

Although over 200 HPV genotypes have been identified, HPV16 is the most prevalent subtype associated with malignancies. It is the primary genotype found in oropharyngeal squamous cell carcinoma (OPSCC), though not the sole variant detected. Variability in genotype distribution likely results from a multifaceted interplay involving viral transmission routes, patterns of exposure, tissue-specific susceptibility, and other yet-to-be-clarified determinants [4,5].

Regional data from Bulgaria underscore the clinical significance of oral HPV screening. A cross-sectional study of dental patients reported oral HPV DNA in 5.1% of oral rinse samples from healthy individuals (predominantly HPV-16) and in 22.9% of neoplastic tissues from patients with oral/oropharyngeal tumors. These findings emphasize the relevance of HPV as a regional health concern and illustrate the importance of population-specific data; however, they also indicate that further epidemiological investigations in Bulgarian cohorts are warranted to fully characterize prevalence patterns and optimize diagnostic approaches [6].

### 1.2. HPV and Oropharyngeal Cancer

HPV is gaining attention in the carcinogenesis of multiple oncological conditions including OPSCC. HPV exerts its carcinogenic effects through multiple mechanisms, including promoting cellular immortalization, inducing genomic instability, impairing DNA damage repair processes, and inhibiting apoptosis [7]. The oncogenic role of HPV in human epithelial tissues is well established, particularly through extensive research on cervical cancer. Robust evidence supports HPV as a key etiological factor, including its integration into the host genome, transcription of viral mRNA, synthesis of viral oncoproteins, and interference with major tumor suppressor pathways. These findings underscore the biological activity of HPV and argue against the idea that its presence in tumors is merely incidental [8].

OPSCC includes malignancies originating from the tonsils, base of the tongue, soft palate, and uvula. In recent years, HPV has been implicated in a significant proportion of these cases—accounting for 71% in the United States and 51.8% in the United Kingdom. Among HPV-positive OPSCCs, 85–96% are linked to HPV16, suggesting that the majority may be preventable through prophylactic vaccination. This vaccine, proven effective in reducing HPV-related cervical lesions, is now routinely administered to both males and females in numerous countries [9,10].

In addition to HPV, other viral infections are also implicated in oropharyngeal squamous cell carcinoma (OPSCC). The International Agency for Research on Cancer (IARC) recognizes Epstein–Barr virus (EBV) as an independent and well-established risk factor for nasopharyngeal carcinoma. In contrast, infection with Herpes Zoster Virus (HZV) may have a protective effect against oral squamous cell carcinoma (OSCC), as shown in a large population-based case–control study, which reported a hazard ratio of 0.41 (95% CI: 0.33–0.50) among individuals with a history of HZV infection [7,11,12].

### 1.3. Influence of Behavioral and Health Factors on the Pathogenesis of OPSCC

Multiple host and environmental cofactors contribute to pathogenesis of OPSCC. Many clinical and epidemiological studies have suggested an association between chronic inflammation and different types of cancer. In humans, 15–20% of the tumors initiate with an inflammatory process [13,14]. The incidence rate of periodontitis increases with age, and similarly, oral carcinoma affects people typically over 40 years of age [15]. In patients with concomitant periodontitis and OC, the tumor microenvironment is likely exposed to pro-inflammatory molecules and numerous periodontal pathogenic bacteria. Thus, periodontitis has been suggested to be involved in the modulation of neoplastic cells [16]. Periodontitis has been linked to many chronic, autoimmune, inflammatory and malignant diseases, including OPSCC [16,17,18].

Contributing factors such as alcohol consumption and tobacco smoking, even passive smoking are considered as potential risk factor in the head and neck squamous cell carcinoma and especially the oropharyngeal SCC [7,19]. Even the exclusive use of smokeless tobacco products—particularly gutka, betel quid with tobacco, and tobacco quid—is strongly associated with a significantly increased risk of head and neck cancers among Indian males, with gutka showing the highest risk. Bidi smoking also independently elevated cancer risk, while exposure to secondhanded tobacco smoke further increased susceptibility, even among non-users. Importantly, switching between different tobacco products did not reduce risk, underscoring the need for public health strategies focused on complete cessation rather than substitution [20,21]. Besides being a major risk factor, continued tobacco smoking significantly increases the likelihood of recurrence and second primary tumors in head and neck squamous cell carcinoma (HNSCC), including oral squamous cell carcinoma (OSCC), and is associated with poorer overall survival. A retrospective case–control study identified tobacco cessation following HNSCC diagnosis as an independent predictor of improved treatment response and long-term survival [22,23].

The transformation of alcohol in acetaldehyde is considered the reason for its carcinogenic effect. Not only its direct effect is considered crucial but genetic mutations affecting the enzymes alcohol dehydrogenase and aldehyde dehydrogenase can impair acetaldehyde metabolism, leading to its accumulation and consequently increasing cancer risk, particularly among individuals with these variants [24,25]. There is increased acetaldehyde production in relation to tobacco smoking which leads to synergistic relations between the two addictions [26]. According to prospective study smoking and/or drinking patients had higher all-cause mortality and oral cancer-specific mortality compared to nonsmoking and non-drinking patients [27].

Other addictions beyond alcohol and tobacco have also been examined in relation to oropharyngeal malignancies. The impact of cannabis on head and neck cancer remains uncertain due to inconsistent findings and its frequent use alongside tobacco. While some studies report no significant link with oral cancer, others suggest a possible dose-dependent risk among heavy users. In contrast, opium use has shown a more consistent association with an increased risk of oral and head and neck cancers, with several studies reporting a significantly elevated risk in affected individuals [28,29,30]. Additional risk factors for oropharyngeal squamous cell carcinoma (OPSCC) include the prolonged use of alcohol-containing mouthwashes, certain occupational exposures, and environmental pollutants such as air pollution, all of which may contribute to increased risk when combined with other lifestyle or genetic factors [31,32,33,34].

### 1.4. Diagnostic Methods for HPV

Recent advancements in HPV detection extend beyond conventional molecular methods, with nanotechnology-based approaches showing enhanced sensitivity, speed, and accuracy. Quantum dot biosensors and electrochemical platforms can detect HPV DNA at nanomolar levels, offering rapid turnaround, high specificity, and potential for point-of-care application. Although still under clinical validation, these technologies hold promise for improving both accessibility and precision in HPV diagnostics. In parallel, comparative studies of next-generation sequencing (NGS), droplet digital PCR (ddPCR), and quantitative PCR (qPCR) have highlighted NGS as the most sensitive and versatile method for early detection of HPV-associated oropharyngeal cancer. NGS not only outperforms qPCR and ddPCR in both plasma and oral rinse samples but also allows the dynamic monitoring of disease progression. Together, these advances mark a significant shift toward more sensitive and non-invasive strategies for HPV detection and monitoring [35,36,37].

Despite the availability of extensive global data, the prevalence and role of HPV in oropharyngeal squamous cell carcinoma (OPSCC) among Bulgarian patients remain insufficiently characterized. Regional epidemiological information is particularly important, as the contribution of HPV to OPSCC can vary widely across different populations due to genetic, behavioral, and environmental factors. Addressing this knowledge gap, the present study investigates the association between HPV infection and OPSCC in a Bulgarian case–control cohort, thereby providing population-specific insights that may support both diagnostic strategies and public health planning.

## 2. Materials and Methods

The study enrolled 50 patients with OPSCC and 39 healthy controls. All participants have agreed to participate in the study and have signed informed consent. Patients were recruited from Alexandrovska Hospital, Pirogov Hospital, and St. Anna Hospital in Sofia, while healthy volunteers were enrolled from the private practices of participating clinicians. Detailed general anamnesis was taken. The local oral status was established in relation to the presence of periodontal disease and local irritating factors including fractured teeth related to the location of the lesion. Smoking habits, including use of electronic cigarettes, alcohol consumption and drugs intake was established. Information about concomitant disease and medication intake was gathered. The sample for HPV was collected via oral rinse samples, brush smear and combined. The use of brush smear and oral rinse samples in our study aligns with molecular HPV detection methods, particularly PCR-based assays for viral DNA. These non-invasive approaches are suitable for identifying HPV presence in exfoliated oral epithelial cells and oral rinse, though they do not distinguish transcriptionally active infections unless combined with mRNA-based assays.

Inclusion criteria were a newly diagnosed and untreated patient with histologically verified primary oropharyngeal squamous cell carcinoma. Patients underwent imaging (CT or MRI) and histological confirmation by biopsy—formalin-fixed, paraffin-embedded tissue. Patients were treated with surgery, radiotherapy, chemotherapy or a combination of these, or were referred for palliative treatment. The control group consisted of volunteers who consented to participate in the study; no matching for age or sex was applied.

In the control group, only limited biological sampling was performed due to feasibility and voluntary participation. Oral fluid and combined brush smear + oral fluid samples were collected exclusively from patients, where these procedures were integrated into the diagnostic process.

In the study the following statistical methods were used:

1. Descriptive statistics
  - Quantitative variables are represented by summary statistical characteristics—arithmetic mean (Mean), standard deviation (SD); minimum and maximum value.
  - Categorical variables are represented by absolute (N) and relative (%) frequencies.
2. One-sample Kolmogorov–Smirnov test to check the shape of frequency distributions for quantitative variables.
3. Chi-square test or Fisher’s exact test—when examining relationships between descriptive (categorical) data with two or more categories.
4. *t*-test—when comparing two independent groups when the distribution of the variable under study is normal.

The adopted significance level is α = 0.05. Statistical significance is accepted when the *p* value is less than α (*p* < 0.05).

The specialized statistical package SPSS (Statistical Package for the Social Sciences) version 20.0 was used to process the data from the study.

The HPV determination was performed with brush smear technique oral rinse method and combined method.

## 3. Results

### 3.1. Descriptive Analysis

**Figure 1:**
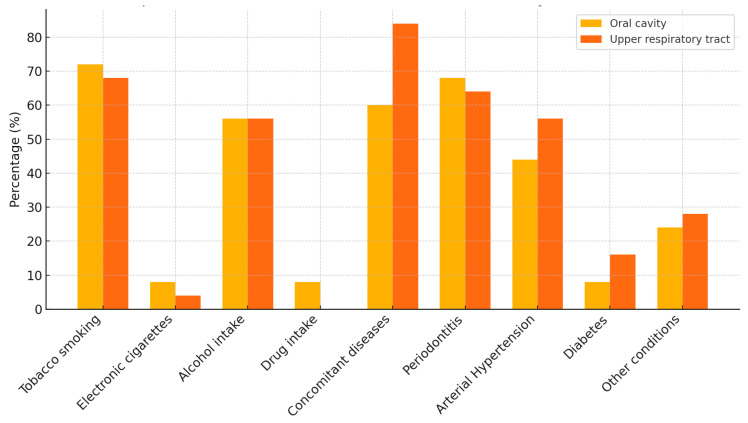
Comparison of risk factors and health conditions by lesion location.

The descriptive analysis regarding the gender distribution age and other important parameters is presented in Table 1 and Table 2. The descriptive analysis regarding the main parameters of the participants is depicted in Figure 1.

A significant difference in gender distribution was observed between the patient and control groups, with males being predominant among patients with oropharyngeal carcinoma (80.0%) compared to controls (41.0%) (*p* < 0.001). This finding aligns with existing literature suggesting a higher prevalence of HPV-associated oropharyngeal cancer in men, potentially reflecting behavioral, immunological, or exposure-related differences between sexes.

No statistically significant differences were observed in the distribution of tobacco smoking, electronic cigarette use, alcohol intake, drug use, periodontitis, or systemic conditions (including hypertension and diabetes) between patients with lesions located in the oral cavity and those in the upper respiratory tract (Figure 1. Appendix A.1). These findings suggest that the evaluated lifestyle and health-related risk factors are similarly represented in both anatomical subgroups and may contribute equally to HPV-associated lesion development across these sites.

### 3.2. Analysis Regarding HPV Findings

There was no significant difference in HPV detection based on gender (*p* = 0.462). Although males were the majority in both HPV-positive and HPV-negative groups, the gender distribution did not differ meaningfully between groups (Table 3). Nevertheless, the observation that over 70% of HPV-positive cases occurred in males aligns with existing evidence suggesting a higher prevalence of HPV-associated oropharyngeal carcinomas in men.

#### 3.2.1. HPV Findings in Oral Location of the Carcinoma

No statistically significant differences were detected between HPV-positive and HPV-negative participants for smoking (75.0% vs. 71.4%; *p* = 1.000), electronic-cigarette use (0% vs. 9.5%; *p* = 1.000), alcohol intake (50.0% vs. 57.1%; *p* = 1.000), or recreational drug use (0% vs. 9.5%; *p* = 1.000). The results are shown in Table 4.

Table 5 presents data on patients with oral cavity tumors, comparing HPV-positive and HPV-negative individuals in relation to several clinical and pathological characteristics. The *p*-values (all = 1.000) indicate no statistically significant associations between HPV status and the evaluated parameters. However, trends suggest that HPV-positive tumors may be more likely to present higher levels of differentiation and ulcer-infiltrative growth patterns.

#### 3.2.2. HPV Findings and Upper Respiratory Tract Location of the Carcinoma

Table 6 shows lifestyle-related risk factors in patients with upper respiratory tract tumors by HPV status. No statistically significant differences were observed (all *p* > 0.05). While alcohol and tobacco use appeared slightly lower in HPV-positive patients, these trends did not reach significance and should be interpreted cautiously.

In upper respiratory tract tumors, no statistically significant differences in clinical or histopathological features were detected between HPV-positive and HPV-negative patients (Table 7). HPV-positive cases more often displayed an ulcer-infiltrative growth pattern and showed a higher frequency of both low and high differentiation, whereas HPV-negative tumors tended to present with exophytic/infiltrative growth and more often exhibited moderate differentiation. These trends, while not significant, suggest potential biological differences that merit further investigation.

### 3.3. Local Factors Related to Oral Manifestations of Carcinoma

**Figure 2:**
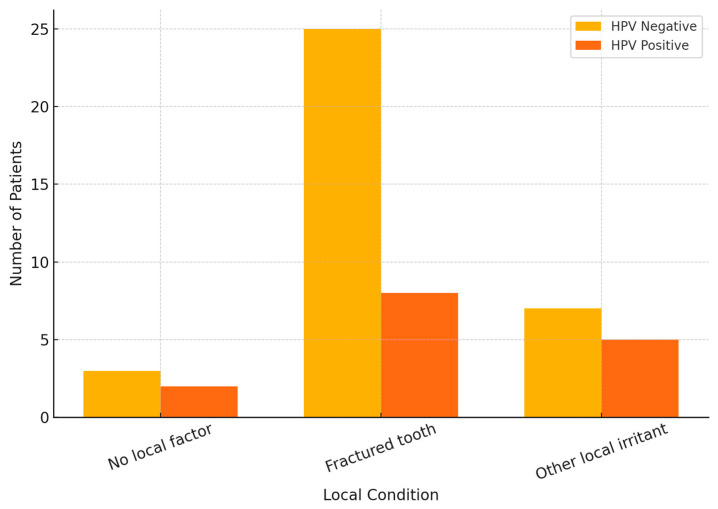
Distribution Of Local Oral Factors By HPV Status.

Analysis of local oral conditions revealed no statistically significant differences between HPV-positive and HPV-negative patients (*p* = 0.452) (Figure 2. Appendix A.2). Fractured teeth were the most common finding, particularly in HPV-negative individuals, while other local irritants appeared slightly more frequent among HPV-positive cases. Although not significant, these trends suggest that chronic local trauma—especially fractured teeth—may contribute to lesion development regardless of HPV status.

### 3.4. Methods for HPV Detection

In the analysis of sample distribution across study groups, most participants—particularly healthy controls—had no positive HPV results (94.9% in controls vs. 70.0% in patients). Brush smear samples were collected in both groups, more often in patients (10.0%) than in controls (5.1%). Among controls, only two HPV-positive cases were detected by brush smear, while no positives appeared with other methods. Oral rinse and combined (oral rinse + brush smear) samples were obtained only from patients (10.0% each). These findings indicate a broader sampling approach in the patient cohort, likely reflecting greater diagnostic or monitoring needs. The results are shown in Table 8.

Among patients with carcinoma, the ulcer-infiltrative growth pattern was the predominant clinical presentation, observed in 80.0% of all cases and across all sample types. It was particularly prevalent in patients from whom smear (100%) or oral rinse/combined samples (80%) were obtained. The exophytic/infiltrative pattern was less frequent (20.0%) and not represented in the brush sample group. Despite these distribution trends, the association between clinical presentation and sample type did not reach statistical significance (*p* = 0.918), suggesting that sample type was not strongly influenced by the tumor growth pattern in this cohort (Table 9).

The distribution of histological diagnoses by sampling method is shown in Table 10. Low to moderately differentiated SCC was predominant (82.0%) and present across all groups, while highly differentiated SCC and other variants appeared only in patients without samples or with oral rinse. The association between tumor differentiation and sample type was not statistically significant (*p* = 0.663), indicating that sampling method was not influenced by histological grade in this cohort.

As shown in Table 11, lesion location did not significantly influence the method of sample collection (*p* = 0.095). Oral cavity and upper respiratory tract lesions were equally represented overall (50.0% each), but combined sampling was performed exclusively in upper respiratory tract tumors. These trends suggest anatomical sites may affect sampling feasibility, although no statistical significance was observed.

### 3.5. Growth Patterns and Histological Types of Carcinomas by Tumor Differentiation

Table 12 shows a significant association between tumor localization and clinical growth pattern (*p* = 0.034). Ulcer-infiltrative lesions predominated overall, particularly in the oral cavity, whereas the exophytic/infiltrative pattern was more frequent in upper respiratory tract tumors. These findings suggest that anatomical sites may influence clinical presentation.

To examine whether tumor location influences histological differentiation, diagnoses were compared between oral cavity and upper respiratory tract carcinomas. Low and moderately differentiated squamous cell carcinoma was significantly more frequent in upper respiratory tract lesions (96.0%) compared to oral cavity tumors (68.0%). Conversely, highly differentiated squamous cell carcinoma and other histological types were observed exclusively or more frequently in oral cavity lesions, with no such cases recorded in the upper respiratory tract. This distribution was statistically significant (*p* = 0.027), suggesting a possible association between anatomical location and histological differentiation of squamous cell carcinoma. The results are summarized in Table 13.

To assess whether tumor differentiation influenced sample collection strategy, the degree of differentiation was analyzed across different sample types (Table 14). Moderate differentiation was the most common overall (70.0%) and was especially dominant in the oral rinse-only group (100%), suggesting a preference or feasibility of collecting oral rinse in tumors with moderate features. In contrast, low differentiation was more frequently observed in patients with combined (60.0%) and smear (40.0%) samples but was absent among those with oral rinse-only specimens. High differentiation remained rare across all groups. The observed differences were statistically significant (*p* = 0.035), suggesting that tumor differentiation may affect or be associated with the type of biological sample collected, possibly due to accessibility, tumor characteristics, or diagnostic prioritization.

## 4. Discussion

Head and neck squamous cell carcinomas (HNSCCs) represent one of the most commonly diagnosed cancers worldwide, ranking sixth in global incidence. The number of new cases has grown considerably over the past two decades, now approaching 900.000 annually, with nearly half as many deaths. This upward trend is projected to continue, with new cases expected to surpass one million per year by 2030. The incidence is particularly elevated in certain economically developed regions, largely due to the increasing prevalence of oropharyngeal cancers (OPC). These malignancies typically originate from the epithelial lining of the oropharynx, including sites such as the soft palate, tonsils, and the rear portion of the tongue. Notably, OPC rates are significantly higher in men—up to five times greater than in women—and in some areas, the incidence among males is nearing, or may soon exceed, that of cervical cancer in females [38,39,40].

Despite technological and therapeutic progress—such as robot-assisted surgeries, multimodal radiation and chemotherapy protocols, and the introduction of immunotherapeutic agents—OPC continues to pose a serious clinical challenge. Many survivors are left with lasting impairments in speech and swallowing, which can severely affect daily functioning and quality of life. Psychological burden is also considerable, with increased rates of depression and suicide observed in this patient population [41].

A range of risk factors have been implicated in the development of HNSCC incl. OPC, including inherited conditions that impair DNA repair, exposure to tobacco and alcohol, environmental carcinogens, and viral infections such as Epstein–Barr virus and human papillomavirus (HPV). Among more than 200 HPV genotypes that infect humans—typically through mucosal contact during sexual activity—a significant portion of the global population will contract the virus at some point. While most infections resolve spontaneously without clinical consequences, persistent infection can lead to diseases such as genital warts, pre-malignant lesions, or cancers of the anogenital region and upper aerodigestive tract. HPV is now recognized as a major contributor to the global cancer burden, implicated in approximately 5% of all cases [40,42].

Epidemiological studies in healthy adult populations from developed nations report variable prevalence of oral HPV, with higher rates in men than in women, and a smaller proportion testing positive for high-risk oncogenic strains. These global trends are consistent with our findings, which also demonstrated a predominance of OPSCC cases among male patients and a rising burden of HPV-associated disease in this subgroup. HPV-16 remains the most prominent causal type in OPC, although other high-risk subtypes such as HPV-18, HPV-31, HPV-33, and HPV-52 may also contribute. The p16 protein is widely used in clinical practice as a biomarker for HPV-related tumors, offering high sensitivity and moderate specificity for identifying HPV-16-driven OPC. Since 2015, many advanced cancer centers have implemented routine p16 testing to support diagnostic accuracy. However, despite mounting evidence linking HPV to the rising burden of OPC, current data from some regions still lack clarity on how many cases are directly attributable to the virus. The present study was designed to address this gap by quantifying the proportion of recent OPC cases driven by HPV. The findings reinforce the increasing incidence of HPV-associated disease and support the broader implementation of gender-neutral vaccination strategies targeting additional high-risk groups [40,43,44].

Previous studies have reported that HPV-positive oropharyngeal squamous cell carcinoma (OPSCC) is more common in individuals without significant tobacco or alcohol use, a history of these risk factors remains prevalent among HPV-positive patients and is linked to poorer outcomes [44,45]. In our study, no statistically significant differences in lifestyle factors—including smoking, alcohol consumption, and e-cigarette use—were observed between HPV-positive and HPV-negative patients. While alcohol use was slightly less common among HPV-positive individuals, this trend did not reach statistical significance. These findings suggest that, within our sample, HPV-related OPSCC occurs independently of traditional behavioral risk factors, though subtle patterns may emerge with larger datasets. Our results are partially consistent with those reported by Lin et al., who examined the correlation between HPV infection and p16 expression in oral and oropharyngeal squamous cell carcinomas [46]. While their study found a moderate concordance between HPV DNA and p16 expression in OPSCC, they also noted that HPV positivity did not always align with specific clinical characteristics. Similarly, in our cohort, no statistically significant associations were observed between HPV status and lifestyle factors such as tobacco, alcohol, or e-cigarette use. These findings reinforce the heterogeneity of HPV-related OPSCC and emphasize the need for further investigation into host and viral factors that may influence its development beyond traditional behavioral risks.

In our cohort, no statistically significant differences were observed between HPV-positive and HPV-negative individuals with respect to smoking, alcohol intake, or recreational drug use. These findings suggest that in our population, HPV status did not correlate with behavioral risk factors, despite the well-established role of such exposures in the pathogenesis of head and neck squamous cell carcinoma (HNSCC). The carcinogenic effect of alcohol is primarily mediated through its metabolism to acetaldehyde, a compound that can induce DNA damage and disrupt cellular repair mechanisms. Genetic variants affecting alcohol dehydrogenase and aldehyde dehydrogenase enzymes further exacerbate this effect by impairing acetaldehyde clearance, thereby amplifying individual susceptibility. Tobacco smoking enhances acetaldehyde production, creating a synergistic effect that magnifies cancer risk when both exposures coexist. Similarly, while the role of cannabis in HNSCC remains debated—owing to inconsistent evidence and frequent concomitant tobacco use—some studies indicate a dose-dependent risk in heavy users. Opium, in contrast, has been more consistently associated with increased oral and oropharyngeal cancer risk, with several reports documenting significantly elevated incidence in exposed individuals. Taken together, although our data did not demonstrate significant differences between HPV-stratified groups, these substances remain clinically relevant cofactors. Their contribution to carcinogenesis likely operates independently of viral status, highlighting the multifactorial nature of oral and oropharyngeal malignancies and underscoring the importance of comprehensive risk assessment in clinical practice.

Our findings reinforce the multifactorial etiology of OPSCC, where HPV-related carcinogenesis may develop independently of traditional behavioral risk factors. This is consistent with the review by Nokovitch et al., which emphasizes that although tobacco and alcohol remain major contributors to OSCC, HPV plays only a limited role in this subsite [30]. They also report increasing OSCC incidence in individuals without established exposures, suggesting the contribution of underrecognized local factors. Similarly, our results showed no significant association between HPV status and lifestyle habits such as smoking or alcohol use, pointing toward additional contributors. Local irritating factors—such as chronic mucosal trauma, inflammation, or poor oral hygiene—may influence susceptibility to HPV infection and progression. These observations are correlational and do not establish causality. Given the cross-sectional design and the potential for confounding by tobacco/alcohol exposure, oral hygiene, and access to care, fractured teeth and local irritants may function as markers of a high-risk milieu rather than independent causes. Reverse causation is also possible, whereby tumor-related changes increase tooth fracture or plaque retention. Prospective and interventional studies that eliminate local irritants and track incident disease are needed to test causality. These elements warrant further investigation to better define risk profiles and guide prevention strategies in HPV-associated OPSCC.

In contrast to oropharyngeal squamous cell carcinoma (OPSCC), where HPV infection is a well-established etiological factor, oral cavity squamous cell carcinoma (OSCC) appears to be more strongly influenced by chronic local irritation. These site-specific exposures include mechanical trauma, poor oral hygiene, and chemical insults from tobacco and alcohol [47]. Our findings support this distinction. Among HPV-negative OSCC cases, fractured teeth were identified in 71.4% of patients, compared to 53.3% in HPV-positive cases, suggesting a stronger association between mechanical irritation and non-viral tumorigenesis. Additionally, other local irritating factors such as prosthetic trauma or mucosal inflammation were found in 20.0% of HPV-negative and 33.3% of HPV-positive cases. Notably, only 10.0% of all cases had no identifiable local trauma or irritant exposure. Although these differences did not reach statistical significance (*p* = 0.452), the high prevalence of fractured teeth and other irritants, particularly in HPV-negative individuals, underscores the critical role of persistent mucosal injury in the development of OSCC. These observations reinforce the concept of distinct pathogenic pathways, wherein OSCC is predominantly driven by chronic local environmental factors rather than viral infection.

Histopathological differentiation is a critical prognostic factor in head and neck squamous cell carcinomas (HNSCC), reflecting tumor aggressiveness and biological behavior. In our cohort, a significant difference was observed between tumor localization and histological grade (*p* = 0.027). Specifically, moderately and poorly differentiated squamous cell carcinomas were predominant in upper respiratory tract (oropharyngeal) tumors (96.0%), while the oral cavity group showed a more diverse pattern, including 16.0% highly differentiated squamous cell carcinomas and 16.0% tumors of other histological types. This suggests that OPSCC tends to present with less differentiated, and potentially more aggressive histological profiles, which may correspond to its frequent association with HPV-driven oncogenesis and distinct molecular pathways. In contrast, OSCC displays greater heterogeneity in differentiation, possibly reflecting the influence of chronic local irritative factors and non-viral etiologies [30,42]. These findings support the notion that anatomical site and underlying etiopathogenesis significantly shape tumor histology, which may have implications for prognosis and treatment strategies.

It is known that histological differentiation plays a critical role in the biological behavior and prognosis of head and neck squamous cell carcinomas. In our study, a statistically significant association was found between tumor localization and histological grade (*p* = 0.027). Specifically, 96.0% of upper respiratory tract tumors were low to moderately differentiated, compared to 68.0% in the oral cavity, where 16.0% were highly differentiated and another 16.0% belonged to other histologic types. This observation highlights a more heterogeneous histopathological landscape in OSCC compared to oropharyngeal tumors. These findings are consistent with the recent review by Tan et al. [48], which emphasized that site-specific etiologic factors—including exposure to local irritants in OSCC versus HPV infection in OPSCC—strongly influence tumor differentiation patterns and biological behavior. Furthermore, the work by de Oliveira et al. [48] demonstrated that while CT texture patterns in OPSCC reflect HPV status, they do not correlate with histological differentiation, suggesting that in HPV-driven oropharyngeal cancers, viral oncogenesis may overshadow the influence of traditional histopathological grading. Taken together, our results reinforce the concept that OSCC and OPSCC represent distinct biological entities, with the oral cavity showing greater histological variability potentially linked to its diverse and chronic local exposures.

Our study shows that tumor differentiation significantly influenced HPV detection (*p* = 0.035), with low-grade tumors predominantly identified through combined sampling (60.0%) and smear-only methods (40.0%) but absent in oral rinse-based testing. Moderate-grade tumors were most prevalent overall (70.0%) and present across all sampling methods, including 100.0% of tumors detected via oral rinse. Highly differentiated tumors were less frequent (10.0%) and appeared sporadically across sample types. These findings align with previous studies reporting that tumor differentiation and anatomical site may influence detection efficiency, likely due to differences in viral load, surface shedding, or viral integration patterns, while others have observed consistent imaging patterns in HPV-related tumors regardless of histological grade [48,49]. A meta-analysis by Gipson et al. found oral rinse testing to offer ~72% sensitivity and ~92% specificity in head and neck cancers but emphasized reduced sensitivity in tumors with poor surface shedding—a likely feature of low-grade or poorly differentiated lesions. Similarly, Castillo et al. (2022) demonstrated that smear-based sampling and liquid-based cytobrush techniques outperform oral rinse in certain settings, particularly in early or keratinized lesions, due to closer proximity to tumor tissue and reduced dilution effects [50,51].

Taken together, our findings emphasize the importance of tailoring HPV detection strategies not only to anatomical site but also to tumor histology. Smear-based or combined methods may offer greater diagnostic yield in low-grade or poorly shedding tumors, while oral rinse testing may be more sensitive in moderately differentiated lesions with higher viral activity. These insights can inform future diagnostic algorithms and support more individualized approaches to HPV testing in head and neck cancers.

Our study has several limitations. The small sample size in certain subgroups, such as HPV-positive cases and combined sample types, limited statistical power. Its cross-sectional design precludes causal inference and outcome analysis. A considerable proportion of participants, especially healthy controls, lacked biological samples, which may have introduced selection bias. HPV detection was binary, without genotype or viral load data, reducing virological detail. Sampling methods varied, potentially affecting consistency. Although some lifestyle factors were recorded, other clinical covariates were not included. Lastly, lesion accessibility may have influenced both sampling feasibility and growth pattern classification, possibly biasing associations with tumor location. Also fractured teeth and local irritants were recorded at diagnosis, reverse causation cannot be excluded, and these variables should be interpreted as associations in this cohort. Despite these limitations, the study provides valuable insights into the interplay between tumor localization, histological differentiation, clinical growth patterns, and sampling feasibility in head and neck carcinoma.

A limitation of our study is that HPV positivity was established solely by DNA detection from oral rinse and brush smears, without assessment of transcriptional activity. Neither E6/E7 mRNA detection nor p16 immunohistochemistry was performed, which are considered surrogate markers of viral oncogenic activity. Therefore, our findings reflect the presence of HPV DNA but cannot fully determine the biological or clinical relevance of the infection. This study has some methodological constraints, including a modest sample size without prior power analysis, voluntary recruitment of controls without demographic matching, and incomplete biological sampling in the control group. Moreover, HPV positivity was assessed only by DNA detection, without markers of transcriptional activity. These factors may limit the strength of certain comparisons, yet the data still provide important exploratory insights into HPV prevalence and clinical patterns in this population. In addition, biological sampling was more limited among controls due to feasibility and voluntary participation, while saliva and combined samples were obtained only from patients within the diagnostic workflow. This imbalance may reduce comparability between groups and should be taken into account when interpreting the results. A further limitation is that genotype-specific distribution, particularly of HPV16, was not assessed in this study; future research will address this aspect to provide deeper insight into viral subtypes and their clinical relevance. The lack of biological samples in a proportion of controls may introduce selection bias and should be considered when interpreting the case–control comparisons.

### Future Directions

Molecular biomarkers, particularly HPV16 E6 antibodies, have demonstrated high specificity and the ability to detect disease years before clinical onset; however, their low prevalence in the general population and the absence of established follow-up protocols reduce their screening utility. Emerging markers, such as HPV circulating tumor DNA, (ctDNA) also show promise for identifying occult or early OPSCC but require validation in large prospective studies. To address current limitations, future research should include larger, demographically balanced cohorts with more systematic biological sampling to overcome underpowered subgroup analyses and improve comparability between cases and controls. In addition, efforts should prioritize refining imaging modalities for better visualization of the oropharyngeal mucosa, enhancing the sensitivity of molecular markers, and defining high-risk populations to enable targeted, early detection strategies for OPSCC [52].

Future research should also explore how tailored sampling strategies—such as integrating saliva and brush-based collection during routine clinical visits or employing non-invasive oral rinses in surveillance protocols—could be realistically embedded into diagnostic and screening workflows for OPSCC.

## 5. Conclusions

The study highlights the influence of anatomical tumor localization on clinical growth patterns and histological differentiation, with statistically significant associations supporting these findings. While HPV status alone did not show strong statistical associations with tumor characteristics, trends in tissue differentiation and local factors suggest underlying biological differences. Additionally, sample type appears to be influenced by both tumor accessibility and differentiation, underscoring the importance of tailoring sampling strategies based on lesion characteristics. Together, these insights enhance our understanding of tumor behavior in head and neck regions and may guide both diagnostic and research methodologies.

## Figures and Tables

**Table 1:** Gender distribution among patients with oropharyngeal carcinoma and control group.

Gender		Group		Total	*p*
		Patients	Control Group		
Male	N	40	16	56	<0.001
	%	80.0%	41.0%	62.9%	
Female	N	10	23	33	
	%	20.0%	59.0%	37.1%	
Total	N	50	39	89	
	%	100.0%	100.0%	100.0%	

*Note: Statistical comparisons were performed using Pearson’s Chi-square test (or Fisher’s exact test when expected cell counts were <5) for categorical variables, and Student’s t-test or Mann–Whitney U test for continuous variables, as appropriate.*

**Table 2:** Average age of the patients and control group.

Parameter	Group	N	Mean	SD	Min	Max
Age	Patients	50	61.57	10.73	38.00	83.00
	Control group	39	49.15	17.02	18.00	77.00

*Note: Statistical comparisons were performed using Pearson’s Chi-square test (or Fisher’s exact test when expected cell counts were <5) for categorical variables, and Student’s t-test or Mann–Whitney U test for continuous variables, as appropriate.*

**Table 3:** Gender-dependent HPV findings.

Parameter			HPV Finding		Total	*p*
			No	Yes		
Gender	Male	N	29	11	40	0.462
		%	82.9%	73.3%	80.0%	
	Female	N	6	4	10	
		%	17.1%	26.7%	20.0%	

*Note: Statistical comparisons were performed using Pearson’s Chi-square test (or Fisher’s exact test when expected cell counts were <5) for categorical variables, and Student’s t-test or Mann–Whitney U test for continuous variables, as appropriate.*

**Table 4:** Comparison of Behavioral Risk Factors in Oral Cavity Carcinoma by HPV findings.

Parameter			Oral Cavity		
			HPV Finding		*p*
			No	Yes	
Tobacco smoking	No	N	6	1	1.000
		%	28.6%	25.0%	
	Yes	N	15	3	
		%	71.4%	75.0%	
Electronic cigarettes	No	N	19	4	1.000
		%	90.5%	100.0%	
	Yes	N	2	0	
		%	9.5%	0.0%	
Alcohol intake	No	N	9	2	1.000
		%	42.9%	50.0%	
	Yes	N	12	2	
		%	57.1%	50.0%	
Drug substances usage	No	N	19	4	1.000
		%	90.5%	100.0%	
	Yes	N	2	0	
		%	9.5%	0.0%	

*Note: Statistical comparisons were performed using Pearson’s Chi-square test (or Fisher’s exact test when expected cell counts were <5) for categorical variables, and Student’s t-test or Mann–Whitney U test for continuous variables, as appropriate.*

**Table 5:** Comparison of Clinical and Histopathological Characteristics in Oral Cavity Tumors According to HPV Status.

Parameter			Oral Cavity		
			HPV Finding		*p*
			No	Yes	
Clinical diagnosis/pattern of growth	Exophytic/Infiltrative	N	2	0	1.000
		%	9.5%	0.0%	
	Ulcer-infiltrative	N	19	4	
		%	90.5%	100.0%	
Histological diagnosis	Low and moderate differentiated squamous cell carcinoma	N	14	3	1.000
		%	66.7%	75.0%	
	Highly differentiated squamous cell carcinoma	N	3	1	
		%	14.3%	25.0%	
	Other	N	4	0	
		%	19.0%	0.0%	
Degree of differentiation	Low	N	2	0	1.000
		%	9.5%	0.0%	
	Moderate	N	16	3	
		%	76.2%	75.0%	
	High	N	3	1	
		%	14.3%	25.0%	

*Note: Statistical comparisons were performed using Pearson’s Chi-square test (or Fisher’s exact test when expected cell counts were <5) for categorical variables, and Student’s t-test or Mann–Whitney U test for continuous variables, as appropriate.*

**Table 6:** Comparison of Lifestyle-Related Risk Factors in Upper Respiratory Tract Tumors According to HPV Status.

Parameter			Upper Respiratory Tract		
			HPV Finding		*p*
			No	Yes	
Tobacco smoking	No	N	4	4	1.000
		%	28.6%	36.4%	
	Yes	N	10	7	
		%	71.4%	63.6%	
Electronic cigarettes	No	N	14	10	0.440
		%	100.0%	90.9%	
	Yes	N	0	1	
		%	0.0%	9.1%	
Alcohol intake	No	N	5	6	0.435
		%	35.7%	54.5%	
	Yes	N	9	5	
		%	64.3%	45.5%	
Drug substances usage	No	N	14	11	n/a

*Note: Statistical comparisons were performed using Pearson’s Chi-square test (or Fisher’s exact test when expected cell counts were <5) for categorical variables, and Student’s t-test or Mann–Whitney U test for continuous variables, as appropriate.*

**Table 7:** Comparison of Clinical Growth Patterns and Histological Differentiation in Upper Respiratory Tract Tumors According to HPV Status.

Parameter			Upper Respiratory Tract		
			HPV Finding		*p*
			No	Yes	
Clinical diagno-sis/pattern of growth	Exophytic/Infiltrative	N	6	2	0.234
		%	42.9%	18.2%	
	Ulcer-infiltrative	N	8	9	
		%	57.1%	81.8%	
Histological diagnosis	Low and moderate differentiated squa-mous cell carcinoma	N	14	10	0.440
		%	100.0%	90.9%	
	Highly differentiated squamous cell carcinoma	N	0	1	
		%	0.0%	9.1%	
	Other	N	0	0	
		%	0.0%	0.0%	
Degree of differentiation	Low	N	3	5	0.136
		%	21.4%	45.5%	
	Moderate	N	11	5	
		%	78.6%	45.5%	
	High	N	0	1	
		%	0.0%	9.1%	

*Note: Statistical comparisons were performed using Pearson’s Chi-square test (or Fisher’s exact test when expected cell counts were <5) for categorical variables, and Student’s t-test or Mann–Whitney U test for continuous variables, as appropriate.*

**Table 8:** Distribution of Sample Types Collected from Patients and Healthy Controls.

Sample		Group		Total
		Patients	Healthy Controls	
No	N	35	37	72
	%	70.0%	94.9%	80.9%
Brush smear	N	5	2	7
	%	10.0%	5.1%	7.9%
Oral rinse	N	5	0	5
	%	10.0%	0.0%	5.6%
Combined	N	5	0	5
	%	10.0%	0.0%	5.6%
Total	N	50	39	89
	%	100.0%	100.0%	100.0%

*Note: Statistical comparisons were performed using Pearson’s Chi-square test (or Fisher’s exact test when expected cell counts were <5) for categorical variables, and Student’s t-test or Mann–Whitney U test for continuous variables, as appropriate.*

**Table 9:** Relationship Between Clinical Growth Patterns and Sample Types in Carcinoma Patients.

Clinical Diagnosis/Pattern of Growth		Sample				Total	*p*
		No	Brush Smear	Oral Rinse	Combined		
Exophytic/Infiltrative	N	8	0	1	1	10	0.918
	%	22.9%	0.0%	20.0%	20.0%	20.0%	
Ulcer-infiltrative	N	27	5	4	4	40	
	%	77.1%	100.0%	80.0%	80.0%	80.0%	

*Note: Statistical comparisons were performed using Pearson’s Chi-square test (or Fisher’s exact test when expected cell counts were <5) for categorical variables, and Student’s t-test or Mann–Whitney U test for continuous variables, as appropriate.*

**Table 10:** Distribution of Histological Diagnoses in Carcinoma Patients According to Sample Type.

Histological Diagnosis		Sample				Total	*p*
		No	Brush Smear	Oral Rinse	Combined		
Low and moderate differentiated squamous cell carcinoma	N	28	5	3	5	41	0.663
	%	80.0%	100.0%	60.0%	100.0%	82.0%	
Highly differentiated squamous cell carcinoma	N	3	0	1	0	4	
	%	8.6%	0.0%	20.0%	0.0%	8.0%	
Other	N	4	0	1	0	5	
	%	11.4%	0.0%	20.0%	0.0%	10.0%	

*Note: Statistical comparisons were performed using Pearson’s Chi-square test (or Fisher’s exact test when expected cell counts were <5) for categorical variables, and Student’s t-test or Mann–Whitney U test for continuous variables, as appropriate.*

**Table 11:** Distribution of Sample Types According to Lesion Location in Carcinoma Patients.

Location		Sample				Total	*p*
		No	Brush Smear	Oral Rinse	Combined		
Oral cavity	N	21	2	2	0	25	0.095
	%	60.0%	40.0%	40.0%	0.0%	50.0%	
Upper respiratory tract	N	14	3	3	5	25	
	%	40.0%	60.0%	60.0%	100.0%	50.0%	

*Note: Statistical comparisons were performed using Pearson’s Chi-square test (or Fisher’s exact test when expected cell counts were <5) for categorical variables, and Student’s t-test or Mann–Whitney U test for continuous variables, as appropriate.*

**Table 12:** Association between tumor growth pattern and anatomical localization in carcinoma patients.

Clinical Diagnosis/Growth Pattern		Localization		Total	*p*
		Oral Cavity	Upper Respiratory Tract		
Exophytic/Infiltrative	N	2	8	10	0.034
	%	8.0%	32.0%	20.0%	
Ulcer-infiltrative	N	23	17	40	
	%	92.0%	68.0%	80.0%	

*Note: Statistical comparisons were performed using Pearson’s Chi-square test (or Fisher’s exact test when expected cell counts were <5) for categorical variables, and Student’s t-test or Mann–Whitney U test for continuous variables, as appropriate.*

**Table 13:** Histological Differentiation of Squamous Cell Carcinoma According to Tumor Location.

Histological Diagnosis		Localization		Total	*p*
		Oral Cavity	Upper Respiratory Tract		
Low and moderate differentiated squamous cell carcinoma	N	17	24	41	0.027
	%	68.0%	96.0%	82.0%	
Highly differentiated squamous cell carcinoma	N	4	0	4	
	%	16.0%	0.0%	8.0%	
Other	N	4	1	5	
	%	16.0%	4.0%	10.0%	

*Note: Statistical comparisons were performed using Pearson’s Chi-square test (or Fisher’s exact test when expected cell counts were <5) for categorical variables, and Student’s t-test or Mann–Whitney U test for continuous variables, as appropriate.*

**Table 14:** Association Between Tumor Differentiation and Type of Biological Sample Collected.

Digree of Differentiation		Sample				Total	*p*
		No	Smear	Oral Rinse	Combined		
Low	N	5	2	0	3	10	0.035
	%	14.3%	40.0%	0.0%	60.0%	20.0%	
Moderate	N	27	2	5	1	35	
	%	77.1%	40.0%	100.0%	20.0%	70.0%	
High	N	3	1	0	1	5	
	%	8.6%	20.0%	0.0%	20.0%	10.0%	

*Note: Statistical comparisons were performed using Pearson’s Chi-square test (or Fisher’s exact test when expected cell counts were <5) for categorical variables, and Student’s t-test or Mann–Whitney U test for continuous variables, as appropriate.*

## Data Availability

All data are available in the article.

## Abbreviations

The following abbreviations are used in this manuscript:

HPV	Human papilloma virus
p53	tumor protein p53
Rb	retinoblastoma protein
OPSCC	Oropharyngeal Squamous Cell Carcinoma
HNSCC	head and neck squamous cell carcinoma

## Appendix A

### Appendix A.1. In Relation to Figure 1

**Parameter**			**Location**		**Total**	***p***
			**Oral Cavity**	**Upper Respiratory Tract**		
Tobacco smoking	No	N	7	8	15	0.758
		%	28.0%	32.0%	30.0%	
	Yes	N	18	17	35	
		%	72.0%	68.0%	70.0%	
Electronic cigarettes	No	N	23	24	47	0.552
		%	92.0%	96.0%	94.0%	
	Yes	N	2	1	3	
		%	8.0%	4.0%	6.0%	
Alcohol intake	No	N	11	11	22	1.000
		%	44.0%	44.0%	44.0%	
	Yes	N	14	14	28	
		%	56.0%	56.0%	56.0%	
Drugs intake	No	N	23	25	48	0.149
		%	92.0%	100.0%	96.0%	
	Yes	N	2	0	2	
		%	8.0%	0.0%	4.0%	
Concomitant diseases and conditions	No	N	10	4	14	0.059
		%	40.0%	16.0%	28.0%	
	Yes	N	15	21	36	
		%	60.0%	84.0%	72.0%	
Periodontitis	No	N	8	9	17	0.765
		%	32.0%	36.0%	34.0%	
	Yes	N	17	16	33	
		%	68.0%	64.0%	66.0%	
Arterial Hipertension	No	N	14	11	25	0.396
		%	56.0%	44.0%	50.0%	
	Yes	N	11	14	25	
		%	44.0%	56.0%	50.0%	
Diabetes	No	N	23	21	44	0.384
		%	92.0%	84.0%	88.0%	
	Yes	N	2	4	6	
		%	8.0%	16.0%	12.0%	
Other	No	N	19	18	37	0.747
		%	76.0%	72.0%	74.0%	
	Yes	N	6	7	13	
		%	24.0%	28.0%	26.0%	

### Appendix A.2. In Relation to Figure 2

**Parameter**			**HPV Finding**		**Total**	***p***
			**No**	**Yes**		
Local status	No	N	3	2	5	0.452
		%	8.6%	13.3%	10.0%	
	Fractured tooth	N	25	8	33	
		%	71.4%	53.3%	66.0%	
	Local irritating factor	N	7	5	12	
		%	20.0%	33.3%	24.0%	
